# Beyond catalyst deactivation: cross-metathesis involving olefins containing *N*-heteroaromatics

**DOI:** 10.3762/bjoc.11.241

**Published:** 2015-11-18

**Authors:** Kevin Lafaye, Cyril Bosset, Lionel Nicolas, Amandine Guérinot, Janine Cossy

**Affiliations:** 1 Laboratoire de Chimie Organique, Institute of Chemistry, Biology and Innovation (CBI)-UMR 8231 ESPCI ParisTech, CNRS, PSL* Research University, 10, rue Vauquelin, 75231 Paris Cedex 05, France

**Keywords:** catalyst deactivation, cross-metathesis, *N*-heteroaromatic, pyridine, ring-closing metathesis

## Abstract

Alkenes containing *N*-heteroaromatics are known to be poor partners in cross-metathesis reactions, probably due to catalyst deactivation caused by the presence of a nitrogen atom. However, some examples of ring-closing and cross-metathesis involving alkenes that incorporate *N*-heteroaromatics can be found in the literature. In addition, recent mechanistic studies have focused on the rationalization of nitrogen-induced catalysts deactivation. The purpose of this mini-review is to give a brief overview of successful metathesis reactions involving olefins containing *N*-heteroaromatics in order to delineate some guidelines for the use of these challenging substrates in metathesis reactions.

## Introduction

**Figure 1:**
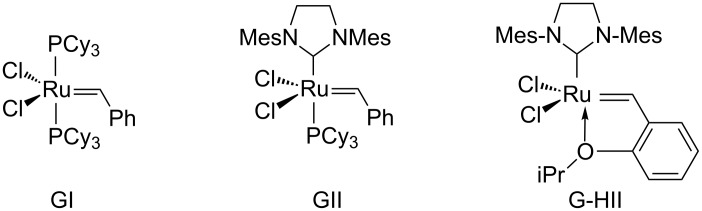
Some ruthenium catalysts for metathesis reactions.

Over the past decades, metathesis has become a key reaction within the organic chemist’s toolbox [1–6]. Since its infancy in the 50’s, metathesis has grown in importance and, today, applications in a broad variety of areas such as natural product synthesis [7–11], polymerization [12], drug discovery [7], petrochemistry or agricultural chemistry have been reported. One of the reasons of this success is the discovery of well-defined, stable, highly chemoselective and now commercially available catalysts particularly the Grubbs catalysts 1^st^ and 2^nd^ generation (GI and GII) and the Grubbs–Hoveyda II catalyst (G-HII) (Figure 1) [13].

A large array of functional groups including alcohols, halides, esters, amides, carbamates and sulfonamides are compatible with the metathesis conditions [14–20]. However, the involvement of alkenes containing a nitrogen atom such as an amine or an *N*-heteroaromatic ring in metathesis reactions is still problematic and have been the subject of several research works [21–26]. Lewis basic and nucleophilic amines are supposed to interfere with the catalyst and/or intermediates, thus disrupting the catalytic cycle and preventing the process to occur (vide infra). Various approaches have been explored to allow the use of primary and secondary amines in ring-closing metathesis (RCM) and cross-metathesis (CM), and one of them is the transformation of amines into carbamates, amides or sulfonamides [27–29]. As an alternative, metathesis reactions can be performed with olefins possessing ammonium salts that can be formed from the corresponding amines either in a preliminary step or in situ, in the presence of an acidic additive [30–35]. In addition, Lewis acids in catalytic amounts were shown to enhance the reactivity of amino compounds in metathesis reactions [36–37]. Involvement of *N*-heteroaromatics containing olefins in metathesis has been less documented. In this review, we would like to give an overview of successful metatheses involving alkenes that possess *N*-heteroaromatics in order to delineate some guidelines. Some mechanistic insights dealing with catalyst deactivation caused by amino derivatives will be first presented and discussed. RCM and CM involving alkenes possessing *N*-heteroaromatics will be then successively examined [38].

## Review

### Mechanistic insights into amine-induced catalyst deactivation

**Scheme 1:**
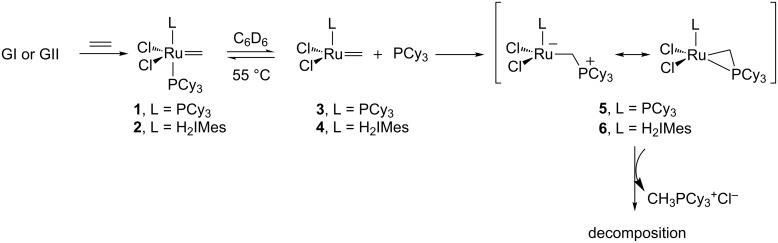
Decomposition of methylidenes **1** and **2**.

Recently, intensive studies dealing with ruthenium catalyst deactivation in metathesis have been published, most of them focusing on the GII catalyst [39–43]. In 2007, Grubbs et al. examined the decomposition pathways of various ruthenium methylidenes using NMR spectroscopy [44]. The methylidenes **1** and **2** derived from GI and GII had a half-life of 40 min and 5 h 40 min, respectively at 55 °C and the main byproduct CH_3_PCy_3_^+^Cl^−^ was identified using ^1^H, ^13^C and ^31^P NMR data as well as HRMS data. The deactivation of the catalysts was hypothesized to go through ligand dissociation from **1** and **2** followed by a nucleophilic attack of the free phosphine on the methylidene intermediates **3** and **4** to give CH_3_PCy_3_^+^Cl^−^ and inactive ruthenium complexes. Similar observations were made in the absence or in the presence of ethylene in the reaction medium (Scheme 1).

**Scheme 2:**
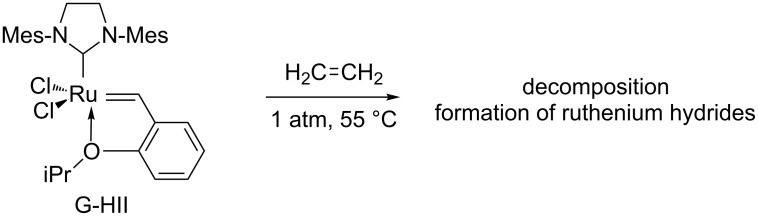
Deactivation of G-HII in the presence of ethylene.

Similar studies concerning the Grubbs–Hoveyda II catalyst were difficult due to the instability of the methylidene derivative that could not be isolated. Thus, the decomposition of G-HII was studied in the presence of ethylene and unidentified ruthenium hydride species were observed by ^1^H NMR after 24 h. This result indicates that another mode of deactivation that does not involve a phosphine is involved in G-HII degradation (Scheme 2).

**Scheme 3:**
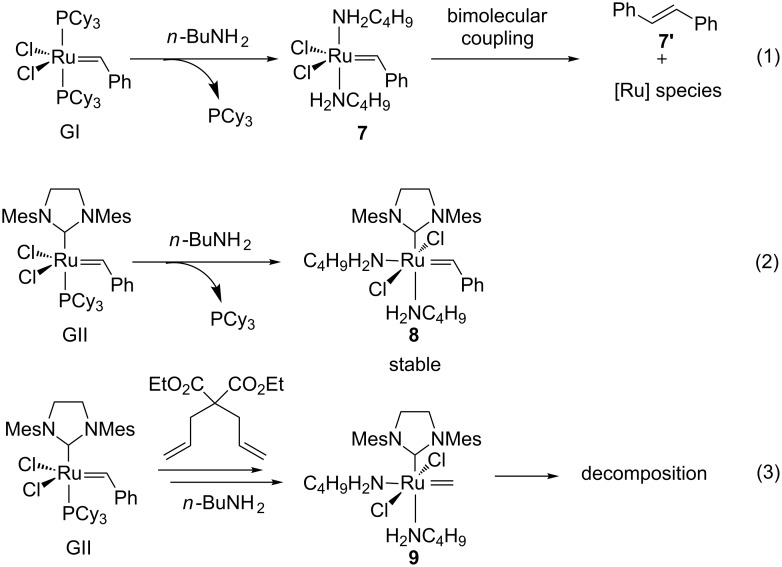
Reaction between GI/GII and *n*-BuNH_2_.

In 2009, Moore et al. studied the stability of GI and GII in the presence of *n*-butylamine using ^1^H and ^31^P NMR spectroscopy [39]. While GI decomposed within 10 min after formation of bisamino complex **7** (Scheme 3, reaction 1), GII resulted in a new stable bis-amino ruthenium complex **8** that was isolated and characterized using X-ray diffraction (Scheme 3, reaction 2). In both cases, free PCy_3_ was observed by NMR confirming amine-induced phosphine displacement. The decomposition of GI was hypothesized to go through a bimolecular coupling from **7**. On the contrary, the bulky NHC ligand present in **8** could prevent this side reaction. However, in the presence of diethyl diallylmalonate and *n*-butylamine, GII decomposed readily probably due to an increased instability of the less hindered methylidene **9** compared to benzylidene **8** (Scheme 3, reaction 3).

**Scheme 4:**
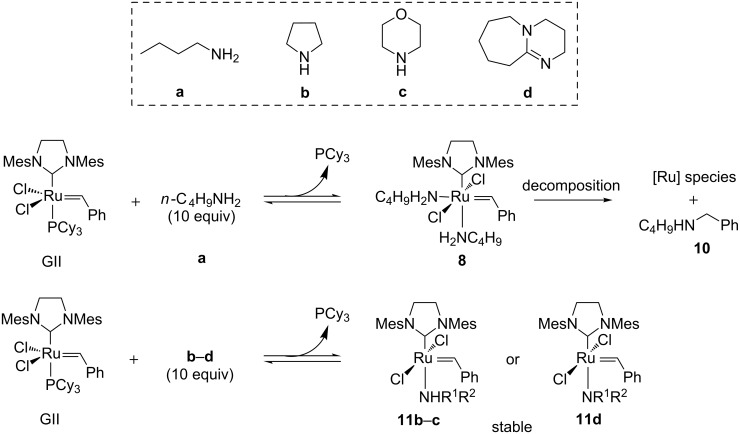
Reaction of GII with amines **a**–**d**.

Fogg et al. completed this study by focusing on amine-mediated degradation of GII and they highlighted various plausible decomposition pathways depending on the nature of the amine [45]. At first, the reaction between GII and various amines such as *n*-butylamine (**a**), pyrrolidine (**b**), morpholine (**c**) and DBU (**d**) were examined by ^1^H NMR. As already highlighted by Moore et al., in the presence of *n*-butylamine, GII was transformed into **8** and the latter slowly decomposed (half-life = 3.5 h) to give ruthenium species and amine **10** as the major identified organic compound. This amine would come from the attack of the non-bulky *n*-butylamine on the hindered benzylidene. With more sterically hindered amines **b**–**d**, the ruthenium complexes **11b**–**d**, resulting from phosphine displacement, proved to be stable even after 24 h at 60 °C (Scheme 4).

The half-life of methylidene **2** derived from GII in the presence of the amines were then evaluated using NMR experiments [45]. The steric hindrance of the amine appeared to be a critical parameter. The non-bulky primary amine *n*-butylamine (**a**) induced a fast decomposition of the methylidene **2** (Table 1, entry 1) whereas secondary amines such as pyrrolidine (**b**) and morpholine (**c**) are less detrimental to the catalyst (Table 1, entries 2 and 3). Interestingly the sp^2^ amine DBU did not induce any decomposition of the methylidene intermediate (Table 1, entry 4).

**Table 1:** Decomposition of methylidene **2** in the presence of amines **a**–**d**.

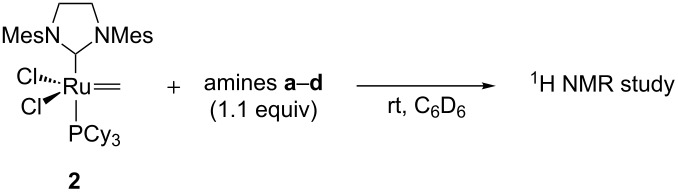		

Entry	Amine	Half-life

1	*n*-C_4_H_9_NH_2_ (**a**)	12 min
2	pyrrolidine (**b**)	1.5 h
3	morpholine (**c**)	14 h
4	DBU (**d**)	>24 h

**Scheme 5:**
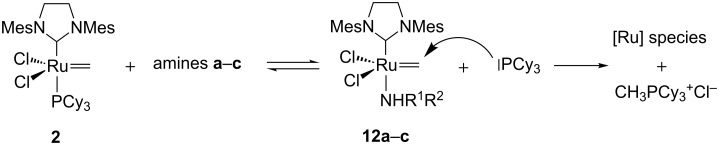
Amine-induced decomposition of GII methylidene **2**.

In all decomposition cases, the main identified product was the phosphonium CH_3_PCy_3_^+^Cl^−^ that would result from a nucleophile attack of the free PCy_3_ liberated through ligand exchange on the methylidene **2** (Scheme 5).

**Scheme 6:**
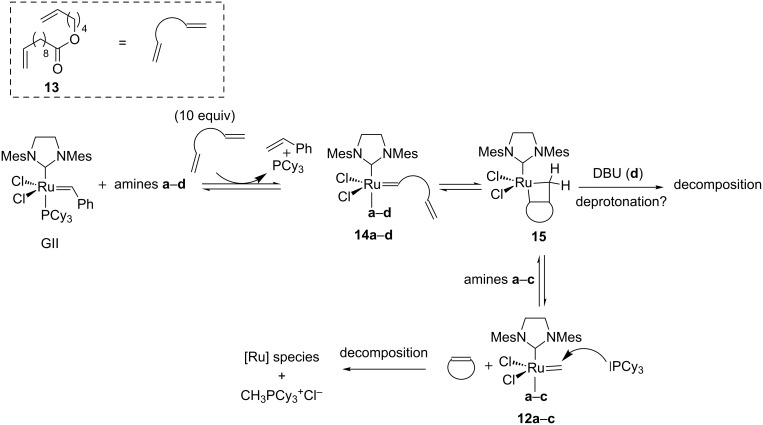
Amine-induced decomposition of GII in RCM conditions.

To complete their study, the authors examined the influence of the amines on the GII-catalyzed RCM of diene **13** [45]. In the presence of amines **a**–**c**, decomposition was observed and CH_3_PCy_3_^+^Cl^−^ was generated. Interestingly, in the presence of DBU, fast decomposition of the catalyst was noticed and only the presence of free PCy_3_ could be observed. According to the previous experiments, DBU was not able to decompose the methylidene resting-state and, consequently, a deprotonation of the metallacyclobutane **15** was hypothesized (Scheme 6).

**Scheme 7:**
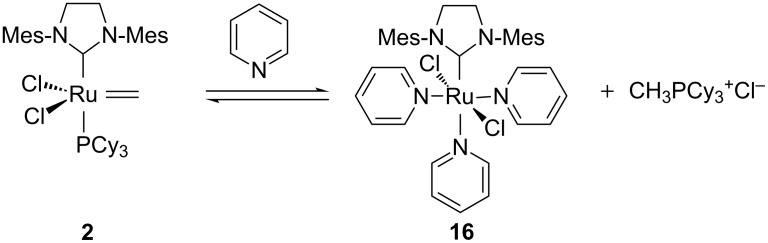
Deactivation of methylidene **2** in the presence of pyridine.

The influence of pyridine as an additive on the deactivation of the metathesis catalyst has not been yet studied in detail [44]. When reacted with an excess of pyridine, the methylidene adduct **2** obtained from GII led to the formation of inactive complex **16** together with CH_3_PCy_3_^+^Cl^−^. These products would result from a ligand exchange followed by a nucleophilic attack of PCy_3_ on the methylidene intermediate (Scheme 7).

**Scheme 8:**
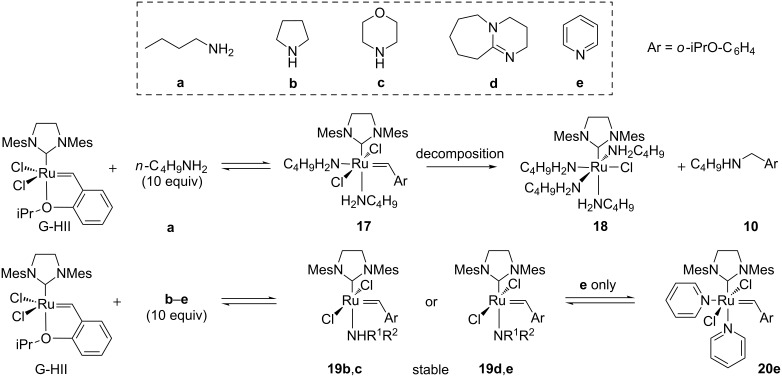
Reaction of G-HII with various amines.

Very recently, the amine-induced deactivation of G-HII catalyst was studied by Fogg et al. [46]. When G-HII was treated with an excess of various amines **a**–**e** (10 equiv), comparable results with those obtained with GII were obtained. In the presence of a non-bulky primary amine such as *n*-butylamine, the bis-aminobenzylidene **17** was formed and complete decomposition was noticed after 12 h at rt yielding ruthenium complex **18** and amine **10**. In the presence of secondary amines **b** and **c** and sp^2^ amine **d**, ruthenium complexes **19b**–**d** possessing one amine were formed and proved to be thermally stable. When the G-HII catalyst was treated with pyridine (**e**), the stable bis-pyridyl adduct **20e** was formed in equilibrium with G-HII and no significant decomposition of the catalyst was observed (Scheme 8).

In contrast, the addition of amino additives such as pyridine, morpholine, Et_3_N or DBU was shown to be detrimental to the G-HII-catalyzed dimerization of styrene (Table 2). Moderate to poor yields in stilbene **7’** were obtained and the value of the yields was correlated with the p*K*_a_ of the couple ammonium/amine. An increased Brønsted basicity of the amine seemed to induce a faster deactivation of the catalyst.

**Table 2:** Impact of amino additives on the CM of styrene.

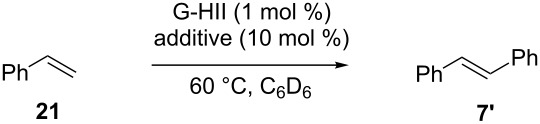			

Entry	Additive	p*K*_a_^a^	Yield

1	none	–	94%
2	pyridine	12.6	45%
3	morpholine	16.6	18%
4	Et_3_N	18.5	9%
5	pyrrolidine	19.6	<5%
6	DBU	24.1	<5%

^a^p*K*_a_ of the conjugate acid in CH_3_CN.

**Scheme 9:**
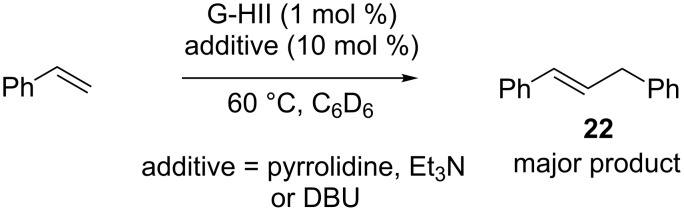
Formation of olefin **22** from styrene.

In addition, when the self-metathesis of styrene was performed in the presence of pyrrolidine, DBU or Et_3_N, olefin **22** was formed as the major product (Scheme 9).

**Scheme 10:**
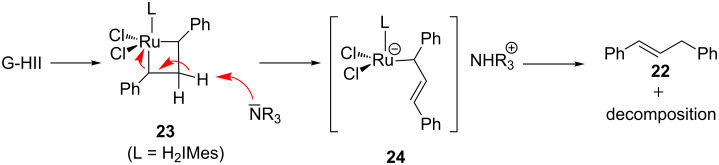
Hypothetic deactivation pathway of G-HII.

To explain these observations, a deactivation mechanism involving a deprotonation of the metallacyclobutane intermediate **23** was hypothesized. The resulting anionic ruthenium complex **24** would be protonated and, after elimination, alkene **22** and unidentified ruthenium complexes would be produced (Scheme 10).

According to these mechanistic investigations, several pathways are involved in the amine-induced catalyst decomposition depending on the nature of the amine and of the ruthenium complex. Non-bulky primary amines can attack directly benzylidene species and are responsible for the fast degradation of the catalyst. In the case of a phosphine-containing catalyst such as GII, secondary amines exchange with PCy_3_ and the free phosphine can perform a nucleophilic attack on the methylidene intermediate triggering its decomposition. In contrast, sp^2^ amines such as DBU seem rather to react with the metallacyclobutane intermediate. In the case of G-HII catalyst, a deprotonation of the metallacyclobutane is hypothesized to explain the amine-induced decomposition (Table 3). Consequently, a modulation of the Brønsted basicity and/or the nucleophilicity of the amine/*N*-heteroaromatic present on an alkene may allow its use in metathesis reactions.

**Table 3:** Amine-induced degradation pathways of GII and G-HII.

	GII	G-HII

Primary amine	Nucleophilic attack on the benzylidene and/or methylidene **2**	Nucleophilic attack on the benzylidene and/or methylidene
Secondary amine	Ligand exchange and nucleophilic attack of free PCy_3_ on the methylidene **2**	Deprotonation of the metallacyclobutane **23**
sp^2^ amine	Nucleophilic attack and/or deprotonation of the metallacyclobutane **15**	Deprotonation of the metallacyclobutane **23**

### Ring-closing metathesis

#### Formation of pyridinium/imidazolium salt prior to metathesis

**Scheme 11:**
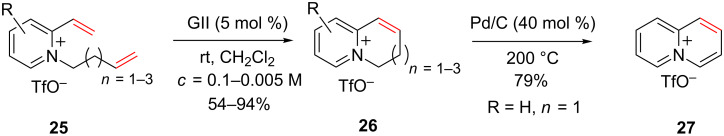
RCM of dienic pyridinium salts.

Most of the examples of RCM involving substrates that possess a pyridine ring relied on the pre-requisite formation of a pyridinium salt. In 2004, Vaquero et al. reported the synthesis of dihydroquinolizium cations through RCM of dienic pyridinium salts in the presence of the GII catalyst (Scheme 11) [47]. The formation of seven- and eight-membered rings required high dilution. Few years later, the same authors showed that it was possible to oxidize 3,4-dihydroquinolizinium salts into their quinolizinium counterparts using Pd/C at high temperature (Scheme 11) [48].

**Scheme 12:**
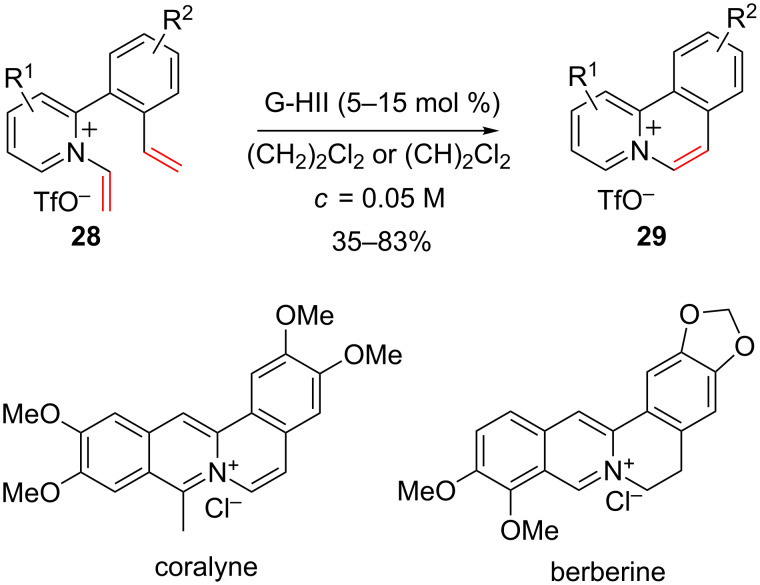
Synthesis of polycyclic scaffolds using RCM.

This method was used to prepare polycyclic scaffolds that can be encountered in diverse alkaloid natural products such as coralyne and berberine (Scheme 12) [49].

**Scheme 13:**
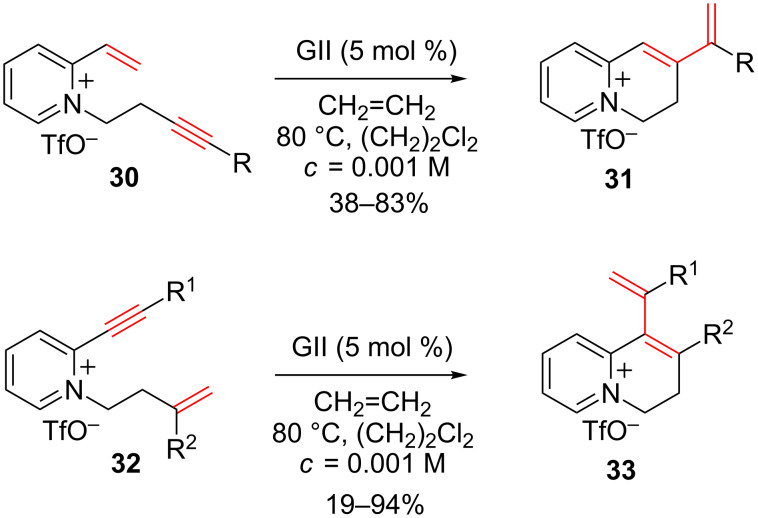
Enyne ring-closing metathesis.

Similarly, enyne ring-closing metathesis reactions were performed to access a variety of vinyl-3,4-dihydroquinolizinium salts (Scheme 13) [50].

**Scheme 14:**
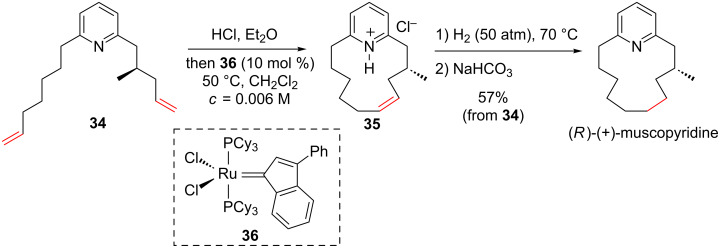
Synthesis of (*R*)-(+)-muscopyridine using a RCM strategy.

In their synthetic approach towards (*R*)-(+)-muscopyridine, Fürstner and Leitner have constructed the 13-membered ring macrocycle using a RCM applied to diene **34** [51]. In order to avoid the catalyst deactivation due to the presence of the pyridine moiety, the precursor **34** was first treated with HCl to form the corresponding hydrochloride salt which was then reacted with the ruthenium catalyst **36** under diluted conditions to deliver **35**. After reduction of the double bond, the targeted (*R*)-(+)-muscopyridine was isolated (Scheme 14).

**Scheme 15:**
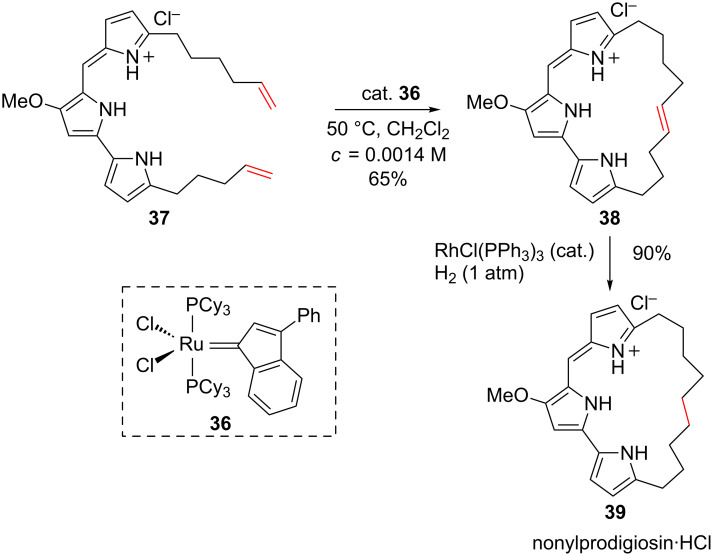
Synthesis of a tris-pyrrole macrocycle.

A similar strategy was used in the synthesis of the tris-pyrrole macrocyclic pigment nonylprodigiosin [52]. A preliminary protonation of the tris-pyrrole followed by a RCM applied to **37** in the presence of the ruthenium catalyst **36** gave the macrocycle **38**, which was then transformed into the saturated derivative **39** using the Wilkinson’s catalyst (Scheme 15).

**Scheme 16:**
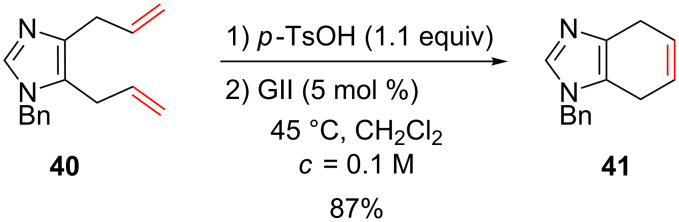
Synthesis of a bicyclic imidazole.

The use of an acidic additive also allowed the synthesis of fused bicyclic imidazoles through a GII-catalyzed RCM reaction (Scheme 16) [53].

**Scheme 17:**
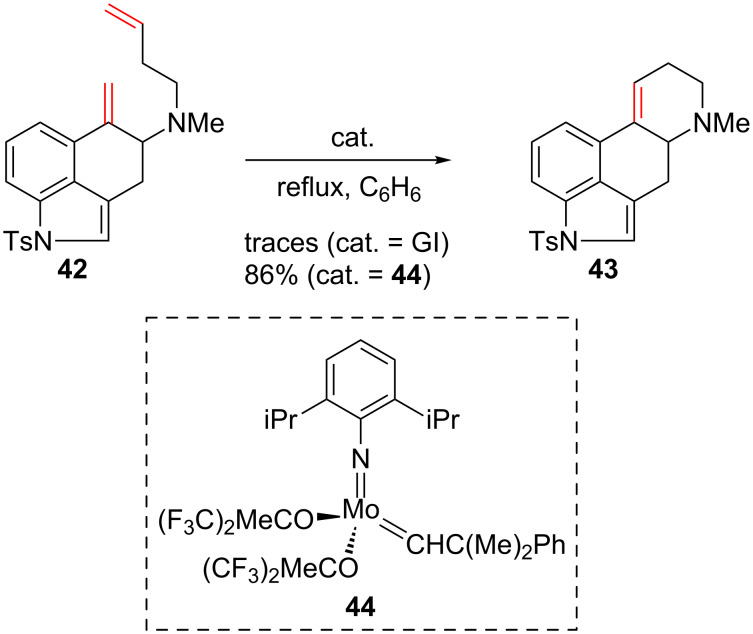
RCM using Schrock’s catalyst **44**.

Only few examples of RCM involving dienes that contain *N*-heteroaromatics were described on non-protonated species. In 2001, in the course of their studies towards ergot alkaloids synthesis, Martin and co-workers used a RCM to form the tetracyclic compound **43** incorporating an indole moiety. A poor yield was obtained in the presence of the GI catalyst and the more reactive Schrock complex **44** had to be used instead. Worthy of note, the indole was protected as a tosylamide and the GI deactivation may be caused by the tertiary amine (Scheme 17) [54–55].

**Scheme 18:**
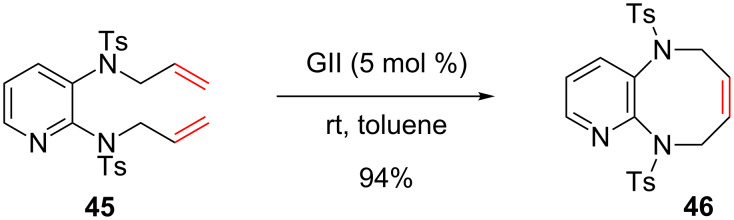
Synthesis of 1,6-pyrido-diazocine **46** by using a RCM.

It should be noted that *N*-heteroaromatics substituted either by bulky or electron-withdrawing groups are involved. In 2004, Billing and co-workers employed a RCM strategy to construct 1,6-pyrido-diazocine **46** with an excellent yield of 94% (Scheme 18) [56]. The presence of the two sulfonamide substituents on the pyridyl ring might decrease the basicity of the nitrogen atom thus allowing the metathesis to proceed. Steric hindrance due to the C2 substitution may also prevent the pyridine-induced catalyst deactivation.

**Scheme 19:**
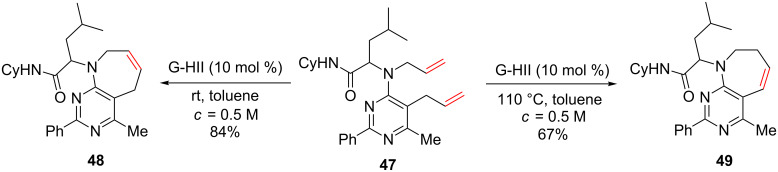
Synthesis of fused pyrimido-azepines through RCM.

Grimaud et al. described the formation of fused pyrimidoazepines from bisallylic substrates using a G-HII-catalyzed RCM [57–58]. When **47** was treated with 10 mol % of G-HII at rt in toluene, the seven-membered ring product **48** was obtained, whereas at 110 °C the isomerized compound **49** was isolated (Scheme 19). It should be noted that in all cases, tetrasubstituted pyrimidines were involved in the RCM and the substituents in the α position of the *N*-heteroatoms might have a role in the success of these reactions by causing steric hindrance around the nitrogen and thus preventing the catalyst deactivation.

**Scheme 20:**
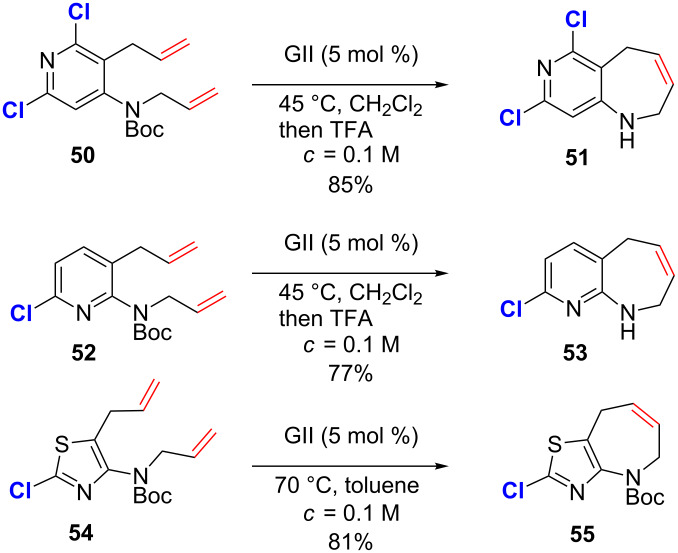
RCM involving alkenes containing various *N*-heteroaromatics.

In 2013, Moss generalized the method to the formation of azepines fused with a variety of heteroaromatics including pyrimidines, pyridines, thiazoles and pyrrazoles [59]. Interestingly, most of the heteroaryls possess a chlorine substituent but no explanation was given concerning its putative role in the success of the RCM (Scheme 20). It should be proposed that the chlorine atoms decrease the basicity of *N*-heteromatics through electron-withdrawing effects and thus reduce the catalyst deactivation. In addition, as chlorine atoms are present in the α position regarding to the nitrogen atom, steric effects cannot be neglected.

**Scheme 21:**
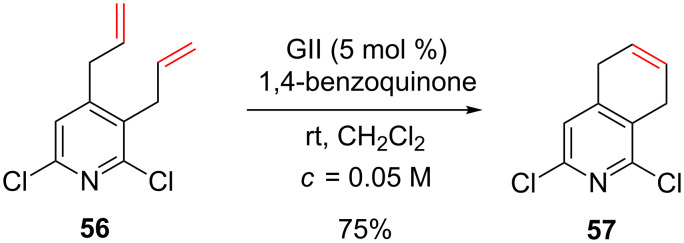
Synthesis of dihydroisoquinoline using a RCM.

Another example of RCM involving alkenes that possess 2-chloropyridines was reported to produce dihydroisoquinoline **57** from 2,6-dichloro-3,4-diallylpyridine (**56**) [60]. The addition of benzoquinone prevented the isomerization of the double bond and it may be suspected that the presence of the two chlorine atoms significantly decreased the basicity of the pyridine (Scheme 21).

**Scheme 22:**
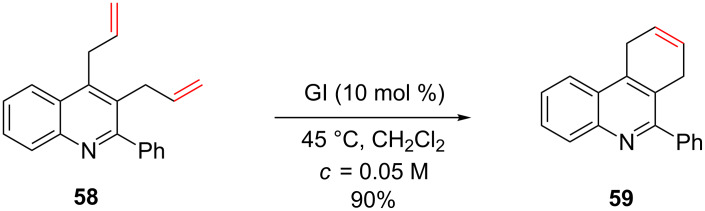
Formation of tricyclic compound **59**.

Tricyclic compound **59** was prepared by a RCM of diene **58** that incorporates a quinoline moiety [61]. In this case, a phenyl group was present at C2 and may be responsible for avoiding the nitrogen-induced deactivation of the catalyst by both electronic and steric effects (Scheme 22).

**Scheme 23:**
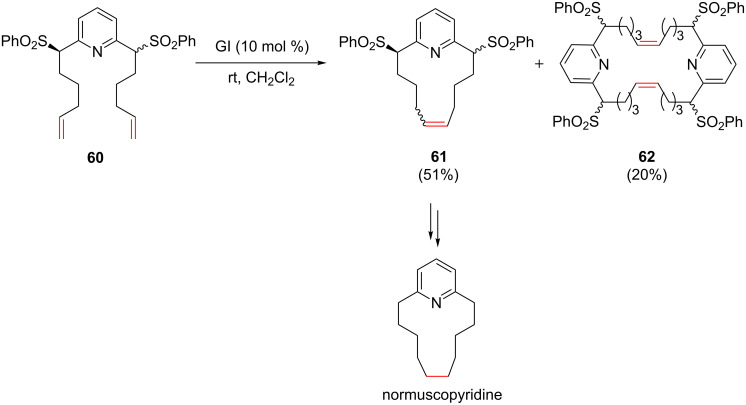
RCM in the synthesis of normuscopyridine.

Macrocycles embedding *N*-heteroaromatics have been prepared using a RCM reaction. Shirbate et al. used a RCM to synthesize normuscopyridine and analogues [62]. When a diastereomeric mixture of 2,6-disubstituted pyridine **60** was treated with GI, the expected macrocycle **61** was obtained (51%) together with the dimeric cyclophane **62** (20%). The authors explained that the sulfone moieties facilitated the RCM by steering the alkenyl chains into a favorable conformation, but it also may be hypothesized that the steric hindrance caused by the sulfone groups might reduce the ability of the nitrogen atom in deactivating the ruthenium catalyst. A desulfonylation followed by a hydrogenation of the double bond afforded normuscopyridine (Scheme 23).

**Scheme 24:**
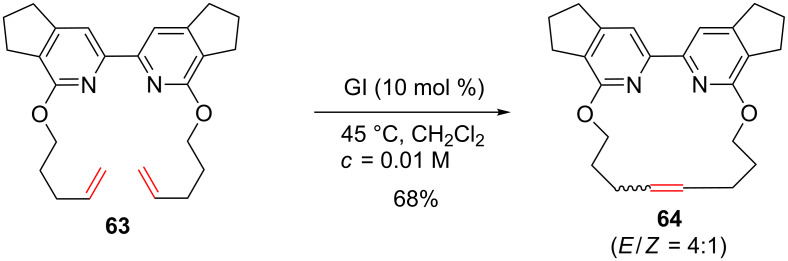
Synthesis of macrocycle **64**.

Other syntheses of cyclophanes using RCM were reported in the literature. Macrocycle **64** was obtained from diene **63** in good yield in the presence of the GI catalyst under diluted conditions [63]. Once again, the presence of the two alkoxy substituents at the C2 position of the pyridyl rings might not be innocent in the success of the RCM and steric hindrance may be invoked to explain the absence of catalyst deactivation (Scheme 24).

**Scheme 25:**
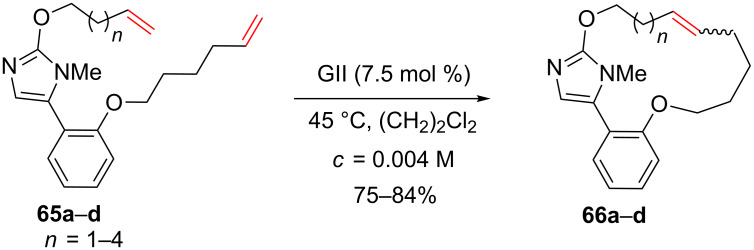
Synthesis of macrocycles possessing an imidazole group.

Similarly, 15- to 18-membered ring macrocycles that incorporate an imidazole group were synthesized using a RCM of the corresponding dienes using GII as the catalyst (Scheme 25) [64–66].

By examining all these examples of successful RCM involving alkenes containing *N*-heteroaromatics, it seems that decreasing their Brønsted basicity and/or their nucleophilicity through the introduction of suitable electron-withdrawing and/or bulky substituents may prevent the catalyst deactivation thus allowing the metathesis to proceed.

#### Cross-metathesis

**Scheme 26:**
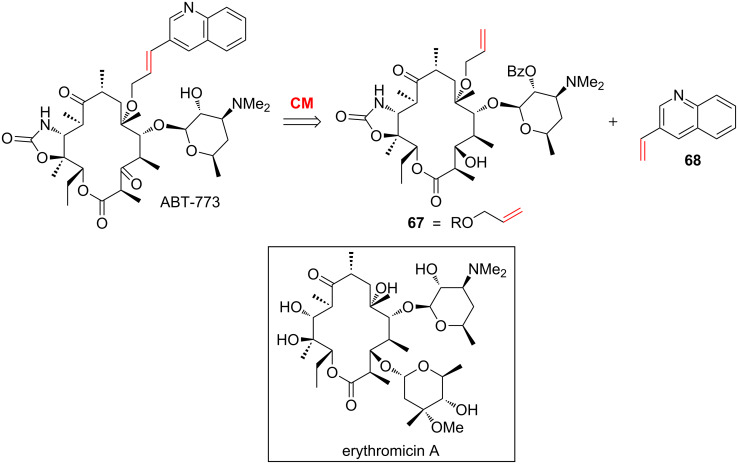
Retrosynthesis of an analogue of erythromycin.

Examples of CM that involve an alkene containing *N*-heteroaromatics as one of the two partners are scarce [67–70]. In 2004, Zhang and co-workers planned to use a cross-metathesis between **67** and vinylquinoline **68** in order to synthesize ABT-773, an analogue of erythromycin possessing a 6-*O*-propenylquinoline side chain (Scheme 26) [71–74].

The cross-metathesis between **67** and vinylquinoline **68** in the presence of the GI catalyst was investigated and the authors showed that the success of the reaction required either long reaction time (168 h) (Table 4, entry 1), high catalyst loading (25 mol %) (Table 4, entry 2) or an excess of the precious macrolide (3 equiv) (Table 4, entry 3). Using an excess of the vinylquinoline **68** (5 equiv) was detrimental to the reaction as **69** was isolated in a poor yield of 23%. This observation might be explained by the deactivation of the GI catalyst caused by the quinoline (Table 4, entry 4).

**Table 4:** CM between vinylquinoline and an *O*-allyl-protected erythronolide derivative.

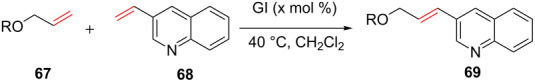					

Entry	**67** (equiv)	**68** (equiv)	Time (h)	GI (mol %)	**69** (yield)

1	1	2	168	10	71%
2	1	2	65	25	75%
3	3	1	65	10	79%
4	1	5	20	10	23%

**Scheme 27:**
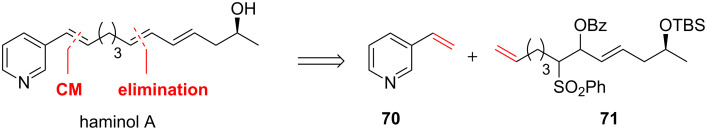
Retrosynthesis of haminol A.

In their retrosynthesis of haminol A, O’Neil et al. initially envisionned to access the trienic compound using a cross-metathesis/benzoyloxysulfone elimination sequence. The CM would involve 3-vinylpyridine **70** as one of the two partners (Scheme 27) [75].

**Scheme 28:**
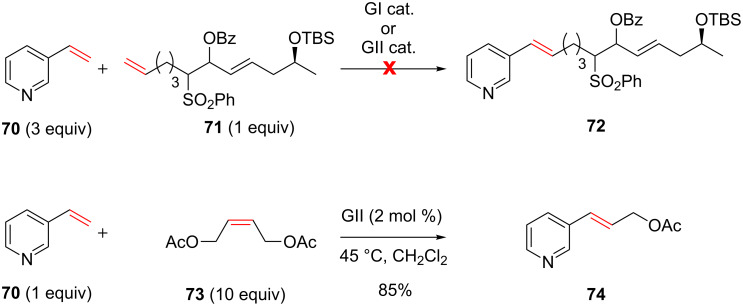
CM involving 3-vinylpyridine **70** with **71** and vinylpyridine **70** with **73**.

As the 3-vinylpyridine **70** was far less precious compared to alkene **71**, it was used in excess in order to favor the CM product over homodimers. However, no reaction occurred neither with GI nor with GII catalysts and the starting materials were recovered. This absence of reactivity was attributed to the deactivation of the ruthenium catalyst due to the excess of pyridine in the reaction medium. Indeed, a successful metathesis was performed between 3-vinylpyridine (**70**) and a large excess of *cis*-1,4-diacetoxy-2-butene (**73**, 10 equiv) delivering the corresponding alkene **74** in 85% yield (Scheme 28).

**Scheme 29:**
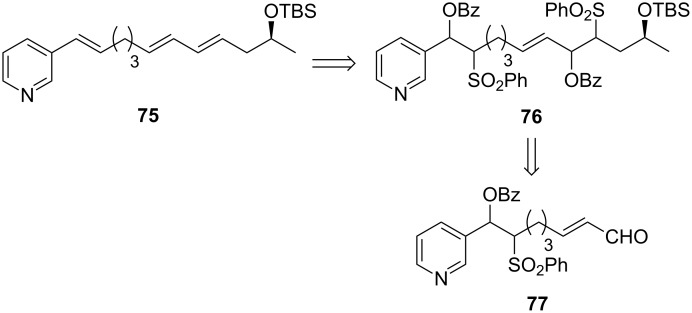
Revised retrosynthesis of haminol A.

As the use of a large excess of the functionalized alkene partner **71** was not attractive, the authors revised their synthetic strategy and finally installed the triene moiety by means of a double benzoyloxysulfone elimination applied to compound **76** which was prepared from aldehyde **77** (Scheme 29).

**Scheme 30:**
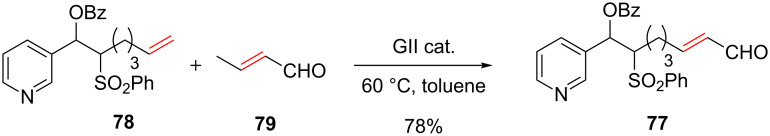
CM between **78** and crotonaldehyde.

Aldehyde **77** was assembled by a CM between alkene **78** and crotonaldehyde (**79**). It should be noted that in this case, the CM proceeded smoothly delivering the desired olefin in 78% yield despite the presence of the pyridine. Worthy of note, the amount of crotonaldehyde added in the reaction was not given in the article (Scheme 30).

**Scheme 31:**
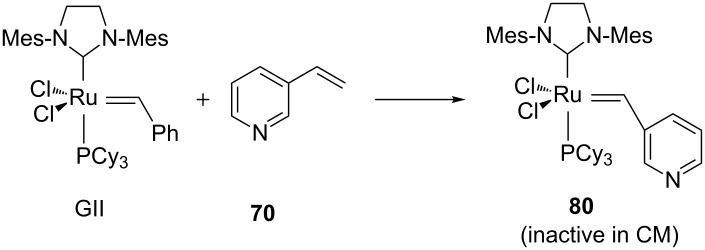
Hypothesized deactivation pathway.

Based on NMR studies, the formation of an inactive ruthenium pyridylalkylidene **80** resulting from a reaction between GII and the vinylpyridine in excess was hypothesized to be the cause of the deactivation of the catalyst (Scheme 31). The use of a large excess of the alkene partner such as *cis*-1,4-diacetoxy-2-butene may statistically prevent the formation of **80** thus allowing the CM to occur.

**Scheme 32:**
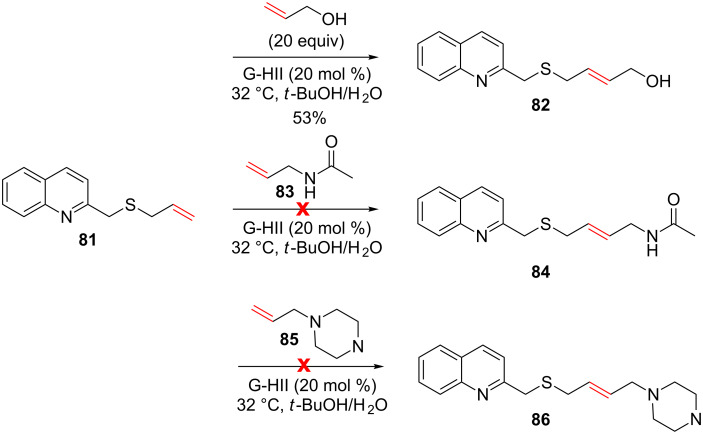
CM involving an allyl sulfide containing a quinoline.

In 2010, Harding et al. attempted to use reversible aqueous metathesis for the construction of a dynamic combinatorial library aimed at identifying DNA ligands [76]. Toward that goal, biologically relevant conditions were selected (rt, *t*-BuOH/H_2_O) and CM involving allyl sulfides that contain functional groups commonly found in DNA-intercalators and *N*-heteroaromatics were investigated. When a quinoline was present on the allylic sulfide, allylic alcohol was found to be the unique suitable partner among the tested olefins. In addition, 20 equiv of allylic alcohol were required and the CM product was obtained in a moderate 53% yield. Cross-metathesis of **81** with amide **83** or alkene **85** gave no conversion (Scheme 32).

**Scheme 33:**
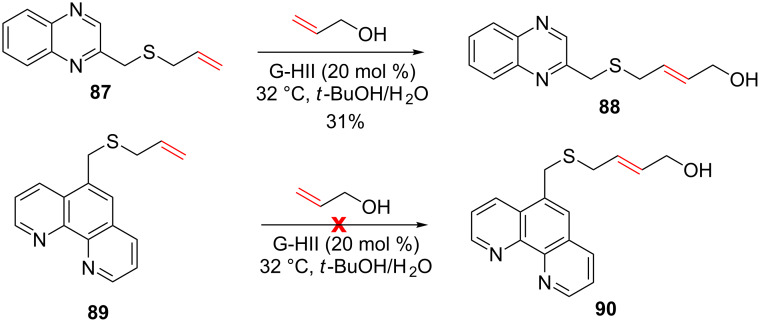
CM involving allylic sulfide possessing a quinoxaline or a phenanthroline.

In the presence of a quinoxaline moiety on the allyl sulfide, the CM reaction with allylic alcohol delivered **88** in a low 31% yield and when an alkene containing a phenanthroline was used, no reaction occurred. By the light of the previously reported observations, these results could be imputed to the deactivation of the ruthenium catalyst caused by *N*-heteroaromatics (Scheme 33).

**Scheme 34:**
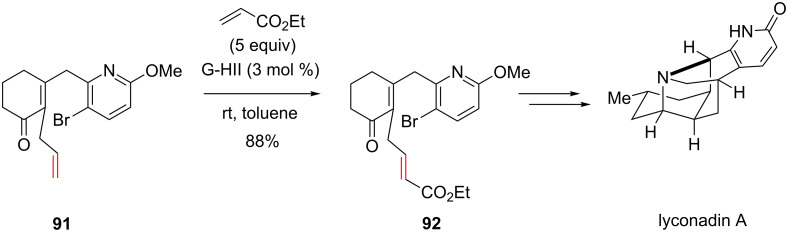
CM between an acrylate and a 2-methoxy-5-bromo pyridine.

One of the rare successful example of CM involving alkene containing a pyridine moiety was reported by Sarpong et al. in their total synthesis of (±)-lyconadin A [77]. Alkene **91** was coupled with ethyl acrylate (5 equiv) using a catalytic amount of the G-HII catalyst to give **92** with a very good yield of 88% (Scheme 34).

**Scheme 35:**
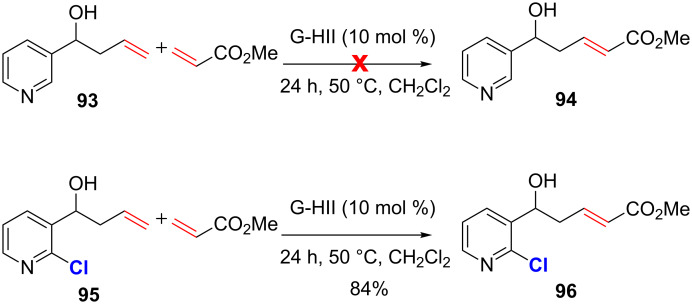
Successful CM of an alkene containing a 2-chloropyridine.

It should be noted that, in this case, the pyridyl ring is substituted by a methoxy group and a bromide and these substituents might be non-innocent in the success of the CM. The presence of 2 substituents at C2 and C6 may cause steric hindrance and the bromine atom at C3 may decrease the basicity of the nitrogen atom through inductive effect. Indeed, in a recent study published by our group, it was demonstrated that successful CM involving alkenes that contain *N*-heteroaromatics could be performed by the introduction of a suitable electron-withdrawing group on the *N*-heteroaryl ring [78]. When olefin **93**, bearing a pyridine without any substituent at C2 or C6, was treated with methyl acrylate in the presence of G-HII catalyst no reaction took place and the starting material was fully recovered. By contrast, the presence of a chlorine substituent at C2 on the pyridyl ring restored the reactivity of the olefin in the CM as the expected product was isolated in 84% yield (Scheme 35). We hypothesized that the presence of the chlorine atom modulates the Lewis and/or Brønsted basicity of the nitrogen atom, thus preventing the deactivation of the ruthenium catalyst (vide infra).

**Scheme 36:**
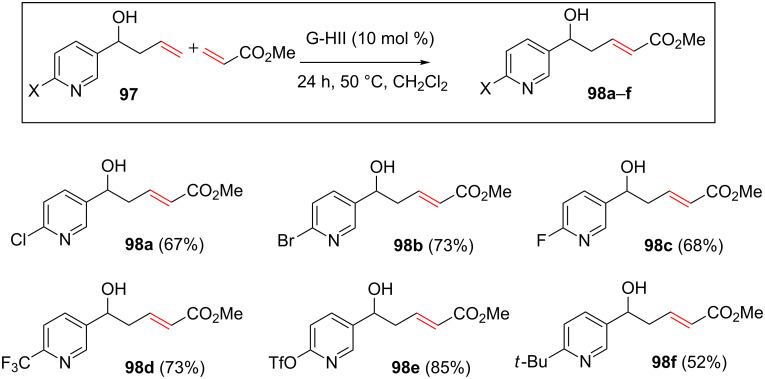
Variation of the substituent on the pyridine ring.

Various substituents on the pyridyl ring such as halides, trifluoromethyl or triflate groups were found to be suitable basicity modulators and the alkenes containing the corresponding disubstituted pyridines were efficiently coupled to methyl acrylate by utilizing a CM reaction. In addition, steric hindrance next to the nitrogen atom could also play a role by decreasing the nucleophilicity of the nitrogen as attested by the formation of alkene **98f** in a moderate 52% yield (Scheme 36).

**Scheme 37:**
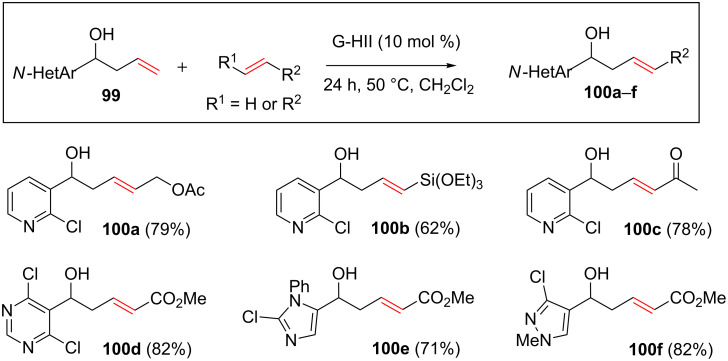
CM involving alkenes containing a variety of *N*-heteroaromatics.

This strategy was applied to the formation of a broad variety of disubstituted olefins containing *N*-heteroaromatic moieties such as pyridines, pyrimidines, imidazoles and pyrazoles (Scheme 37).

From the selected examples discussed above, we tried to delineate some trends regarding to the use of alkenes possessing *N*-heteroaromatics in RCM and CM. In RCM, GI and GII are usually preferred and diluted conditions are recommended to avoid dimerization. The most studied strategy allowing the use of olefins bearing *N*-heteroaromatics is the formation of the *N*-heteroaromatic salt prior to metathesis. The salt can be either isolated before metathesis or formed in situ using acidic additives. Alternatively, introduction of bulky and/or electron-withdrawing substituents on the *N*-heteroaromatic ring allows the metathesis to proceed by preventing nitrogen-induced catalyst deactivation. However, no general study dealing with the influence of the *N*-heteroaromatic substituents on the outcome of the RCM has been led so far. In CM, G-HII may be considered as the most potent catalyst even if some examples involving GII catalyst are described in the literature. Two strategies can be adopted to use olefins possessing *N*-heteroaromatics as one of the partner. When the second partner is non-expensive, it can be introduced in large excess thus avoiding the *N*-heteroaromatic induced catalyst deactivation. As an alternative, bulky and/or electron-withdrawing substituents can be introduced on the *N*-heteroaromatic to reduce the basicity of the nitrogen atom and thus the deactivation. This strategy appears as the most promising especially as a simple chlorine substituent is sufficient to allow the metathesis to proceed (Table 5).

**Table 5:** Metathesis involving alkenes that contain *N*-heteroaromatics.

Metathesis	Cat.	Conditions	Strategies

RCM	GI or GII	diluted	* *N*-heteroaromatic salt formation prior to CM
CM	G-HII		* Non *N*-heteroaromatic partner in large excess * Bulky and/or electron withdrawing substituent on the *N*-heteroaromatic

## Conclusion

*N*-Heteroaromatics are known to have a deleterious impact on metathesis by inducing ruthenium catalysts deactivation. Based on NMR and kinetic mechanistic studies, Lewis and/or Brønsted basicity of amines appeared to be responsible for the degradation of the catalyst. The most common solution proposed to circumvent the problem is the protonation of the nitrogen atom of *N*-heteroaromatics prior to the metathesis that can then be carried out using the corresponding salts. By close examination of the successful metatheses involving alkenes that possess non-protonated *N*-heteroaromatics, the presence of electron-withdrawing and/or bulky substituents on the heteroarene was noticed to be beneficial. These substituents can allow a fine tuning of the basicity and/or nucleophilicity of the nitrogen thus preventing the catalyst deactivation. By unravelling catalyst deactivation pathways, mechanistic investigations could help to extend the scope of the metathesis reactions to alkenes containing *N*-heteroaromatics, thus overcoming one major barrier to the widespread use of metathesis, particularly for industrial purposes.
